# Outcomes of a Foldable Capsular Vitreous Body Implantation: A Retrospective Study

**DOI:** 10.1155/2021/6575195

**Published:** 2021-12-26

**Authors:** Xiangzhong Xu, Huimin Ge, Jiajun Li, Weihong Shang, Yuke Ji, Weihua Yang, Keran Li

**Affiliations:** Department of Ophthalmology, The Affiliated Eye Hospital of Nanjing Medical University, Nanjing 210029, China

## Abstract

**Background:**

The vitreous body is an important part of the ocular body fluid. A foldable capsular vitreous body (FCVB) is designed to treat chronic adverse complications in severe ocular trauma and silicone oil-dependent eyes. This study is aimed at investigating a method for implanting an FCVB, its postoperative efficacy, and clinical value.

**Methods:**

A retrospective analysis was performed on data from 18 patients who underwent vitrectomy and FCVB implantation for severe ocular trauma and silicone oil-dependent eyes between March 2019 and May 2020. All treated eyes underwent clinical examinations involving the best-corrected visual acuity, intraocular pressure, FCVB position, anterior segment photography, and wide-angle fundus photography regularly after surgery.

**Results:**

Eighteen eyes from 18 patients were enrolled in this study. A total of 2.00–4.20 (3.46 ± 0.78) ml of silicone oil were injected into the FCVB during surgery. The patients were followed up at 1, 2, and 4 weeks and 3, 6, and 12 months after surgery. Twelve months after surgery, visual acuity improved in 7 (38.89%) eyes. In contrast, 10 (55.56%) eyes showed no obvious improvement, and 1 (5.56%) eye had decreased vision. Intraocular pressure at 12 months was 10.13 ± 3.52 mmHg, which was comparable to that before the surgery (*t* = 0.38, *P* = 0.71). The anterior chamber depth examined by slit lamp was 2.00–3.00 cornea thickness (CT) in 7 eyes, 1.00–2.00 CT in 2 eyes, and <1.00 CT in one eye. The anterior chamber disappeared in eight eyes. There were eight eyes with clear cornea, four eyes with localized opacity, and two eyes with obvious gray-white opacity. There was no case of severe FCVB deflection, rupture, or exposure during the observation period.

**Conclusion:**

FCVB implantation is an effective and safe treatment for eyes with severe ocular trauma and silicone oil-dependent eyes. It may support retinal reattachment, slow down eyeball atrophy, reduce the risk of chronic adverse complications such as corneal endothelial decompensation, and maintain intraocular pressure and preoperative visual function.

## 1. Introduction

The vitreous body is an important part of the ocular body fluid, which supports the retina, supplies nutrition, stabilizes intraocular metabolism, and acts as a cell barrier and ocular refractive media [1]. Furthermore, the natural vitreous body is nonrenewable. Severe ocular trauma, complex retinal tears, and proliferative diabetic retinopathy can cause damage to intraocular tissues and reduce ocular function; common chronic complications include eyeball atrophy and corneal endothelial decompensation. In recent years, vitreoretinal surgery combined with vitreous tamponade, which includes sterile gas (air, expandable gas), liquid (balanced salt solution, silicone oil (SO), and perfluorocarbon liquid, among others), and polymer (hydrogel), has been used to replace the natural vitreous body with vitreous substitutes to support the retina, promote the anatomical reduction of the retina, and prevent eyeball atrophy. However, nearly 18% of patients remain at risk of ocular atrophy after SO removal [2]. In addition, long-term retention of SO in the eye can lead to secondary glaucoma, cataracts, keratopathy, anterior chamber oil emulsification, and other complications [3]. Some patients need to replace SO repeatedly, which results in severe physical and financial burdens. As a new vitreous substitute product, a foldable capsular vitreous body (FCVB) has an anatomical structure comparable to that of the human vitreous cavity. It is mainly composed of three parts: the vitreous cavity, drainage tube, and drainage valve. The vitreous cavity is an elastic film formed by the polymerization of polyvinyl siloxane and polymethylhydrosiloxane by computer simulation, and the capsule wall is approximately 0.01 mm thick. The drainage valve is connected to the vitreous body through a drainage tube with a diameter of approximately 1.00 mm, which has excellent mechanical properties, optical properties, and biocompatibility that help support the retina [4]. Compared with other established vitreous substitutes used in the clinic, the FCVB remains stable in the eye for a long time, while avoiding serious complications caused by SO filling [4]. However, at present, FCVB has not been promoted in clinical application, and the number of cases in previous studies was also low. In addition, the clinical safety and efficacy of FCVB have not been verified yet. In this study, FCVB implantation, as a new method for treating severe ocular trauma or SO-dependent eyes, was performed in 18 patients to analyze the safety and effectiveness of the FCVB and to evaluate its clinical value.

## 2. Materials and Methods

### 2.1. Study Design

A retrospective study of 18 patients (17 male and 1 female) that underwent FCVB implantation between March 2019 and May 2020 was performed. The patients' average age was 50.61 ± 11.63 years (range, 34–70 years). Fourteen eyes with severe eyeball rupture were treated with phase I debridement and suturing, among which 10 eyes were treated with FCVB implantation within 5–14 (11.5 ± 5.21) days after the injury, and 4 eyes were treated with the same approach within 1.5–4 (2.88 ± 1.03) months after the injury. FCVB implantation was performed in the other four SO-dependent eyes due to vitrectomy and SO filling for severe ocular trauma.

The inclusion criteria were as follows: (1) severe ocular rupture with retinal and choroid injury, visual acuity below 0.05; (2) ocular trauma with mild ocular atrophy, ocular axis of 16.00–28.00 mm; (3) SO-dependent eye after vitrectomy, with secondary complications such as band-shaped degeneration of the cornea, glaucoma, or SO emulsification.

Exclusion criteria were as follows: (1) severe liver and kidney dysfunction, cardiovascular disease, nervous system disease and other systemic diseases, and inability to tolerate FCVB implantation; (2) single eye; (3) severe intraocular infections, uveitis, or intraorbital infections; (4) diseases affecting the development of the orbit in the contralateral eye; (5) allergies to silicone materials or scars; (6) ineligibility for FCVB implantation; (8) overall health unsuitable for participation in a clinical trial; (9) poor compliance and inability to complete the test process as required.

This study followed the principles of the Declaration of Helsinki and was approved by the Ethics Committee of the Affiliated Eye Hospital of Nanjing Medical University (20210015). All patients and their families were informed of the undertaken precautions and possible complications related to the surgery as well as the FCVB implant before the surgery, and an informed consent form was signed by each participant. The clinical trials strictly adhered to the principles of the World Medical Association's Declaration of Helsinki.

### 2.2. Surgical Technique

**Figure 1:**
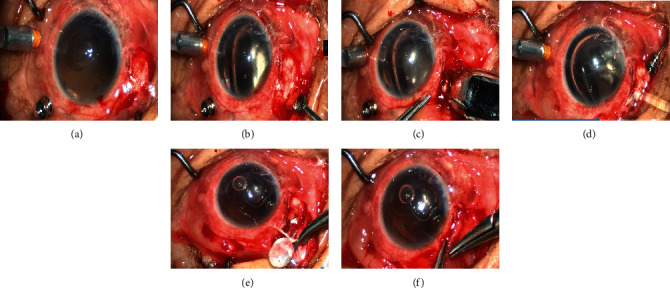
Surgical FCVB implantation. (1) A three-channel incision was made in the supranasal, supratemporal, and inferotemporal regions posterior to corneoscleral margin. (2) An L-shaped scleral incision of approximately 4 mm was made 5 mm posterior to the corneal limbus. (3) FCVB was folded and implanted into the eyeball cavity through the incision with a push injector, and the lens surface of the capsule was placed facing the lens. (4) SO was slowly injected into the capsule through the drainage valve with a syringe until the IOP was moderate. (5) The scleral incision was sutured, and the drainage tube was ligated and fixed in the sclera. (6) Contrapuntal suture of conjunctiva was performed.

All operations were performed by the same surgeon. (1) A three-channel vitrectomy was conducted to remove the central and peripheral vitreous, SO, and vitreous hemorrhage, peeling off the surface or subretinal proliferative membrane. (2) An approximately 4 mm incision was performed on the sclera as FCVB implantation site at either the 4 or 8 o'clock position 5 mm away from the corneal limbus. (3) Perform gas-fluid exchange to lift the IOP over 50 mmHg and evaluate the state of retina and cornea. (4) A suitable type of capsule was prepared preoperatively according to the length of the patient's ocular axis, while the capsular integrity was checked and the scleral puncture port was enlarged to 4 mm. (5) An FCVB was properly folded and implanted into the eyeball cavity through the incision with a push injector. The lens surface of the FCVB was placed facing the lens. If there was any tilting, the position of the FCVB capsule was adjusted with an iris repositor. Subsequently, the SO was slowly injected into the capsule through the drainage valve with a syringe until the intraocular pressure (IOP) was moderate, while the conditions of the retina and color of the optic disc were observed with the aid of an optical fiber. (6) The scleral incision was sutured, and a drainage tube was ligated and fixed to the sclera (Figure 1).

After the surgery, patients were strictly instructed to lie in a prone position for 1 month. Standard postoperative medication: (1) systemic administration: antibiotics, dexamethasone, and hemostatic, followed by prednisone (0.6 mg/kg orally, decreasing weekly) for 8 weeks; (2) local medication: tobramycin and dexamethasone eye drops and ointment (2 months, according to the condition of the eye); cycloplegia; (3) the treatment time of severe eye trauma and children patients should be extended to 3-4 months; (4) avoid strenuous exercise and overuse of eyes.

### 2.3. Follow-Up Examinations

Measurement of best-corrected visual acuity and IOP (NIDEK, NT-5100), corneal endothelial cell test (NIDEK, CEM-530), UBM (SUOER, SW-3200L), ocular axis test (Optical Biometry, IOLMaster 700), B-ultrasound (Meda Co, ltd, ODM-2100S), anterior segment photography (cqsaikang, LS-6), fundus photography (Panoramic Ophthalmoscope-Daytona, A10600), facial photography (NICON, IXUS145), and orbital computed tomography examination (SomATom 90) were performed for all patients before surgery. Best-corrected visual acuity, intraocular pressure, FCVB position, anterior segment photography, and fundus photography were performed at 1, 2, and 4 weeks and 3, 6, and 12 months after the implantation.

### 2.4. Statistical Analysis

In this study, SPSS 23.0 statistical software was used for data analyses. Descriptive statistics were presented as frequencies and composition ratios, and measurement data were expressed as mean ± SD. The *t*-test was used for comparisons of normally distributed binocular parameters before and after surgery; the Wilcoxon matched-pair signed-rank test was used for the analysis of nonnormally distributed data. Count data were compared using the chi-square test. Statistical significance was set at *P* values of <0.05.

## 3. Results

### 3.1. General Condition

**Figure 2:**
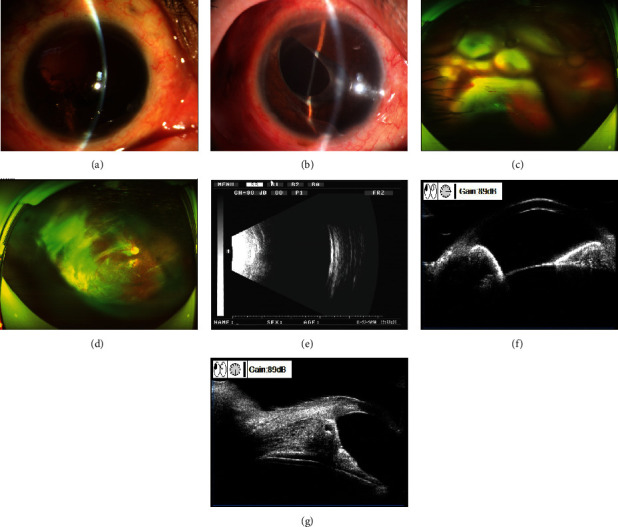
Examination results before and 12 months after FCVB implantation in stage II eyeball rupture. (a) Preoperative anterior segment photography revealed that the vitreous cavity was filled with SO, and the SO moved into the anterior chamber. The cornea was clear; most of the iris and crystal were absent. (b) Anterior segment photography at 12 months after surgery revealed that the cornea was clear, the anterior chamber was not shallow, part of the iris and the lens were absent, and FCVB was in place in the vitreous cavity. (c) Preoperative fundus photography showed retinal folds and extensive retinal detachment. (d) Fundus photography at 12 months after surgery displayed that the retina was flat. (e) B-ultrasound at 12 months after surgery showed the reflection of the posterior wall of the eyeball as smooth, and the FCVB shape as intact. (f, g) UBM at 12 months after surgery showed approximately normal anterior chamber depth, with opened chamber angle, and smooth front surface of FCVB.

**Figure 3:**
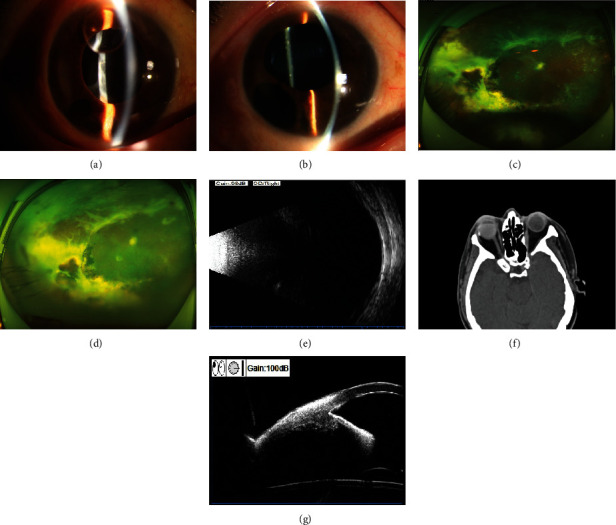
Examination results before and 12 months after FCVB implantation in the SO-dependent eye. (a) Preoperative anterior segment photography indicated that the vitreous cavity was filled with SO, and the SO was moved into the anterior chamber. The cornea was clear, the pupil was dilated but had upward deviation, and the lens was absent. (b) Anterior segment photography at 12 months after surgery revealed that the cornea was clear, the pupil was still round but with upward deviation, and iris root incision was unobstructed. (c) Preoperative fundus photography showed extensive degeneration, detachment, and proliferation of peripheral retina lesions. (d) Fundus photography 12 months after surgery indicated that the retina was flat. (e) B-ultrasound eye at 12 months after surgery showed the reflection of the posterior wall of the eyeball as smooth and the FCVB morphology as intact. (f) Postoperative orbital computed tomography displayed that the appearance of eyeball was normal, the FCVB position was correct, and the SO was filled in place. (g) UBM at 12 months after surgery showed approximately normal anterior chamber depth, with opened chamber angle and smooth front surface of FCVB.

An FCVB was successfully implanted in 18 eyes of 18 patients in this study. The amount of SO injected into the FCVB was in the range of 2.00–4.20 (3.46 ± 0.78) ml. During the follow-up period, no FCVB rejection, displacement, rupture, exposure, sympathetic ophthalmitis, bullae keratopathy, or other serious surgical complications were observed in any patient. Postoperative findings are presented in Figures 2 and 3.

### 3.2. Vision

Twelve eyes (66.67%) had no light sensation before surgery, 3 (16.67%) eyes had preserved light sensation, and 3 (16.67%) eyes had manual sensation. Twelve months after the operation, 8 (44.44%) eyes had no light sensation, 3 (16.67%) eyes had preserved light sensation, and 7 (38.89%) eyes had manual sensation. Among them, there were 7 (38.89%) eyes with improved vision, 10 (55.56%) eyes without obvious improvement, and 1 (5.56%) eye with decreased vision.

### 3.3. Intraocular Pressure

Preoperative IOP was in the range of 3.00–30.00 (10.17 ± 6.82) mmHg in all subjects. The IOP was in the range of 3.00–26.00 (11.44 ± 6.24) mmHg 1 week after surgery, which represented a significant change from baseline (*t* = −0.66, *P* = 0.52). Twelve months after surgery, the mean IOP of the other 16 eyes was 10.13 ± 3.52 mmHg, except for two eyes in which IOP could not be measured due to corneal degeneration. The IOP of 6 eyes was of <8.00 mmHg and that of 12 eyes was in the normal range (8.00–21.00 mmHg). No patient had IOP of >22.00 mmHg, and these values did not change from baseline (*t* = 0.38, *P* = 0.71).

### 3.4. Conditions of Anterior Chamber and Capsule

Twelve months after surgery, 7, 2, 1, and 8 eyes had anterior chamber depth of 2.00–3.00 CT, 1.00–2.00 CT, <1.00 CT, and undetectable value, respectively. No patient had any obvious deviation in FCVB, with intact morphology and no capsular rupture or exposure. Thin exudate membranes were found on the anterior surface of the implanted capsule or pupil area in four eyes, and YAG laser resection was performed.

### 3.5. Corneal Condition

All enrolled patients underwent the corneal endothelium count and corneal thickness assessment before surgery. Corneal endothelium and corneal thickness could not be assessed in 9 and 10 patients, respectively, due to a large amount of anterior chamber hematoma and corneal opacity before surgery. The corneal endothelium count of the remaining nine patients was in the range of 1554.00–2710.00 (2113.00 ± 443.09) CD/mm^2^ and in the range of 2441.00–3046.00 (2690.22 ± 186.644) CD/mm^2^ for contralateral eyes. This difference was statistically significant (*t* = −3.77, *P* = 0.005). The corneal thickness of eight eyes was in the range of 475.00–754.00 mm (627 ± 81.983) mm and in the range of 468.00–591.00 (534.5 ± 36.516) mm for the contralateral eyes. This difference was statistically significant (*t* = 3.11, *P* = 0.02).  Table 1 shows the corneal opacity before and after FCVB implantation in 14 patients with severe ocular rupture. Twelve months after the surgery, the cornea remained clear in eight eyes; among them, the FCVB was implanted in six eyes at stage I and two eyes at stage II post-injury. There were four eyes with localized corneal opacity, including two eyes with FCVB implantation in stage I and two eyes with FCVB implantation in stage II post-injury. There were two eyes with total corneal opacity (gray and white) with FCVB implantation in stage I post-injury (both patients had severe corneal injury, ciliary body injury, low intraocular pressure, and mild atrophy before surgery). The other four silicone oil-dependent eyes had no significant change in corneal opacity before and after FCVB implantation.

## 4. Discussion

The vitreous body helps maintain IOP, support normal anatomical structure of intraocular tissues, and stabilize intraocular metabolism. Severe ocular trauma is a kind of complex disease, often accompanied by cornea, sclera, iris, lens, vitreous body, retina, and choroid injury. Fiber blood vessels and complexity of glial hyperplasia caused low intraocular pressure after retinal detachment and ciliary body damage, leading to long-term chronic complications such as loss of vision, eyeball atrophy, and even enucleation of the eyeball. Therefore, vitrectomy is an effective way for the treatment of severe eye trauma. Currently, vitreous substitutes have some serious shortcomings, such as short duration of intraocular maintenance, high toxicity, and many complications. Precisely, there is no perfect material that has the ability to completely mimic the functions of natural vitreous body along [1, 4].

FCVB was designed to change the traditional supporting mode of the retina with a 360° arc solid pressure effect and isolated the SO with the capsule of FCVB, which is a brand-new therapy strategy. Since that, FCBV implantation has greatly reduced the incidence of complications. Furthermore, patients were no need to keep prone posture after surgery. Silicone oil can also be extracted through the drainage valve of the FCVB or injected with normal saline and silicone oil to regulate intraocular pressure [5, 6].

This study included 10 patients who underwent vitrectomy combined with FCVB implantation and SO injection at stage I (an average of 11.5 days) after debridement of severe eyeball rupture. Severe tissue damage such as massive hemorrhage of the vitreous body, total retinal detachment and defect, and choroidal detachment with cyclodialysis were observed during the surgery. Four patients underwent SO removal combined with FCVB implantation and SO reinjection at stage II (an average of 2.9 months) after debridement for severe ocular trauma. Mild retinal atrophy was observed before surgery, and severe retinal detachment occurred again after the removal of the emulsified SO during surgery. There were four cases of SO-dependent eyes, i.e., repeated silicone oil injection and removal, and the course of disease ranged from 8 months to 10 years. These cases may have become SO-dependent eyes, accompanied by different degrees of eyeball atrophy. Traditional SO filling has poor postoperative efficacy; therefore, FCVB combined with SO injection should be considered.

Twelve patients without light perception before surgery were enrolled in this study, while four patients had restored visual acuity including light perception or hand moving after the surgery, and more than 50% of the patients maintained preoperative visual acuity. Lin et al. found that FCVB implantation could not effectively improve postoperative visual acuity in patients with severe retinal detachment and severe macular scarring, choroid detachment, or other ocular tissue injuries before surgery but could maintain the preoperative visual acuity [5]. Meanwhile, FCVB implants had no significant effect on the diopter of the Gullstrand-Emsley model eyes before and after surgery [6, 7]. *In vivo* studies revealed that FCVB implantation could promote anatomical reduction of the detached retina, specifically, in patients whose lower retina could not be restored after SO filling [5]; it could also inhibit the migration of activated neurogliocytes, collagen fiber IV, and microglial cells into the vitreous cavity during retinal healing [8]. These studies indicate that FCVB implantation combined with SO filling can effectively restore the retina for a long time and promote partial recovery of visual function. For patients with severe ocular injury, preoperative visual function can be maintained even if postoperative visual acuity cannot be improved. However, one patient had no light sensation after surgery, which was thought to be associated with severe retinal detachment and scarring caused by acute retinal necrosis. Animal studies have shown that FCVB implantation can effectively simulate natural vitreous morphology and maintain normal IOP in the short and long term after surgery [7, 9]. At the last follow-up assessment, 14 (77.78%) eyes had an IOP of more than 10 mmHg; two patients could not be examined due to severe corneal degeneration. Measurements in the remaining 16 eyes showed no significant change from baseline values, indicating that FCVB implantation could effectively control further ocular atrophy and this finding is consistent with that of a study by Yanni et al. [5, 10]. Moreover, FCVB implantation can prevent the displacement of SO in the vitreous cavity, reduce the occurrence of secondary ocular hypertension caused by obstruction of the anterior chamber angle or crystal turbidity expansion, promote the recovery of ciliary body function to maintain the stability of aqueous fluid circulation [11], and effectively delay the progression of ocular atrophy. However, some studies have shown that retinal structural disorders were found 180 days after FCVB filling [9], suggesting that we need to explore and find an ideal target intraocular pressure to alleviate the mechanical damage caused by long-term FCVB filling.

Chen et al. showed that anterior chamber depth did not change significantly 6 months after FCVB implantation compared with that before surgery [8]. However, anterior chamber disappearance occurred in eight patients during the follow-up period; meanwhile, no significant increase in IOP was observed in our studies, which may be associated with severe ocular trauma resulting in ciliary body detachment, ciliary body dysfunction, and decreased aqueous fluid secretion function [12]. In addition, some studies have shown that in FCVB implantation, ciliary body reduction and suturing are feasible for patients with ciliary body separation; for patients with traumatic aniridia, polypropylene suture can be used to intercept and suture the ciliary sulcus, which can effectively reduce the occurrence of postoperative superficial anterior chamber and corneal degeneration [13]. For patients with a shallow anterior chamber after surgery, a viscoelastic agent injection can effectively restore the position of iris-FCVB, slow down the drainage of the aqueous humor, and promote the formation of anterior chamber.

Overall, 11%–49% of patients experience SO displacement and nearly all patients experience SO emulsification 12 months after traditional vitrectomy, resulting in secondary glaucoma, corneal degeneration, and other serious complications [14]. In our studies, the corneas remained clear in eight eyes at 12 months of follow-up; however, the corneas were gray and white with obvious opacity in two eyes with severe ocular rupture and there was no significant change in this parameter compared with its preoperative values. These findings suggest that FCVB implantation effectively reduces the occurrence of corneal degeneration caused by long-term SO dependence. Early FCVB implantation reduces SO damage to the corneal endothelium. Nevertheless, due to persistent low IOP caused by early posttraumatic ciliary body hypofunction and intraocular inflammation, shallow anterior chamber and decompensation of the corneal endothelium are likely to occur. For the patients undergoing FCVB implantation in phase II, intraocular inflammation was effectively controlled, and no obvious eyeball atrophy was observed.

FCVB implantation combined with SO refilling can further promote the recovery of intraocular tissue structure and function [15]. In addition, it can be used as an intraocular drug sustained-release system (for drugs such as dexamethasone, levofloxacin, and 5-fluorouracil) in a time- and dose-dependent manner that directly acts on retinal tissues, improves drug bioavailability, increases drug action duration, and inhibits intraocular inflammation [16–18]. Therefore, FCVB implantation effectively reduces corneal endothelial decompensation, persistently low IOP, and helps prevent chronic complications such as progressive eyeball atrophy. However, it was not a comparative study and lack of long-term follow-up. To ensure the superiority and safety of FCVB among all the vitreous substitutes, it is certainly worth conducting large-scale comparative clinical trials in the future.

## 5. Conclusions

The present findings suggest that FCVB implantation can be effective for patients with severe ocular trauma and SO-dependent eyes. It can support the retina, maintain eyeball shape, and stabilize visual acuity, which may become a new carrier of intraocular fluid that integrates vitreous substitutes and drug sustained-release systems, providing a new direction for the treatment of traumatic and refractory vitreoretinal diseases. However, the cost of FCVB is expensive and longer follow-up periods of observation are deficient. Future studies should include larger samples and longer follow-up periods to validate the present findings.

## Figures and Tables

**Table 1:** Corneal opacity condition before and after FCVB implantation for ocular trauma.

Corneal condition	Before surgery			12 months after surgery		
	Phase I for FCVB implantation group	Phase II for FCVB implantation group	In total	Phase I for FCVB implantation group	Phase II for FCVB implantation group	In total
Still clear	6	2	8	6	2	8
Localized opacity	4	2	6	2	2	4
Total opacity	0	0	0	2	0	2

## Data Availability

All the data supporting our findings is contained within the manuscript and tables. The relevant raw data will be freely available from the Affiliated Eye Hospital of Nanjing Medical University by request.

## Abbreviations

FCVB: Foldable capsular vitreous body
SO: Silicone oil
IOP: Intraocular pressure
CT: Cornea thickness.
